# Transient Neonatal Diabetes Mellitus in SHORT Syndrome: A Case Report

**DOI:** 10.3389/fped.2021.650920

**Published:** 2021-06-23

**Authors:** Shin-Hee Kim, Minsung Kim, Jisook Yim, Myungshin Kim, Dae-Hyun Jang

**Affiliations:** ^1^Department of Pediatrics, Incheon St. Mary's Hospital, College of Medicine, The Catholic University of Korea, Seoul, South Korea; ^2^Department of Laboratory Medicine, College of Medicine, The Catholic University of Korea, Seoul, South Korea; ^3^Catholic Genetic Laboratory Center, Seoul St. Mary's Hospital, College of Medicine, The Catholic University of Korea, Seoul, South Korea; ^4^Department of Rehabilitation Medicine, Incheon St. Mary's Hospital, College of Medicine, The Catholic University of Korea, Seoul, South Korea

**Keywords:** SHORT syndrome, PIK3R1 gene, transient neonatal diabetes mellitus, insulin resistance, dysmorphic feature

## Abstract

SHORT syndrome is a rare autosomal dominant disorder characterized by multiple congenital defects and is historically defined by its acronym: *s*hort stature, hyperextensibility of joints and/or inguinal hernia, ocular depression, Rieger anomaly, and teething delay. Herein, we report a male infant with SHORT syndrome who presented with transient neonatal diabetes mellitus (TNDM) with insulin resistance. The proband was born at 38 weeks of gestation but displayed facial dysmorphic features. Intrauterine growth restriction (IUGR) was detected on a prenatal ultrasonography test. His birth weight was 1.8 kg (<3rd percentile), length 44 cm (<3rd percentile), and head circumference 31 cm (<3rd percentile). The patient's blood glucose level started to increase at 5 days of age (218–263 mg/dl) and remained high at 20 days of age (205–260 mg/dl). He was treated with subcutaneous insulin and the blood glucose level gradually stabilized. Blood glucose level was stabilized over time without insulin treatment at 6 weeks of age. Clinical exome sequencing showed a heterozygous pathogenic variant, NM_181523.3:c.1945C>T (p.Arg649Trp) in exon 15 of the phosphoinositide-3-kinase regulatory subunit 1 (*PIK3R1*) known as the causative gene for SHORT syndrome. Examination of the patient at 10 months of age revealed no hyperglycemic episode and glycated hemoglobin level was 5.2%. To the best of our knowledge, this is the first case of TNDM in SHORT syndrome due to a pathogenic variant of *PIK3R1*. We believe that our case can aid in expanding the phenotypes of SHORT syndrome.

## Introduction

SHORT syndrome is a rare genetic disorder with features that include *s*hort stature, hyperextensibility of joints and/or inguinal hernia, ocular depression, Rieger anomaly, and teething delay (1). Additional clinical features include intrauterine growth restriction (IUGR), partial lipodystrophy, inguinal hernia, sensorineural hearing loss, and facial characteristics such as triangular face, small chin, low set posteriorly rotated ears (2, 3). SHORT syndrome was first described in 1975 and to date, fewer than 50 cases have been reported in the literature (2, 3). SHORT syndrome is caused by a heterozygous loss of function in a variant of phosphoinositide-3-kinase regulatory subunit 1 (*PIK3R1*) gene. *PIK3R1* codes for the regulatory subunits of the phosphatidyl inositol-3 kinase of class IA (PI3K) and is involved in the activation of the AKT/mTOR pathway to ensure proper growth and cell proliferation. A pathogenic variant of *PIK3R1* in SHORT syndrome disrupts not only IGF-1R signaling but also insulin signaling and thereby predispose the individual to insulin resistance and diabetes mellitus. It reduce its insulin-stimulated activity that leads to lower AKT and mTOR phosphorylation (4–6). Reduced insulin signaling may also limit intrauterine growth, usually resulting in very small infants at birth (<3rd percentile) (7).

In the present study, we report an infant with transient neonatal diabetes mellitus (TNDM) with insulin resistance who was finally diagnosed with SHORT syndrome due to a pathogenic variant in *PIK3R1*.Written informed consent was obtained from the parents for the publication of this case report.

### Case Report

The patient was a newborn male and the only child of a healthy non-consanguineous Korean couple with a non-contributory family history. The height of his father and mother was 170 cm (−0.70 SD score) and 160 cm (−0.04 SD score), respectively. They had no dysmorphic features. The mother had regular antenatal check-up and did not have any history of medical and obstetric problems during pregnancy. He was born at 38 weeks of gestation but displayed features of IUGR during pregnancy. His birth weight was 1.8 kg (<3rd percentile), length 44 cm (<3rd percentile), and head circumference 31 cm (<3rd percentile) according to the Korean reference for birth weight based on gestational age and sex. The initial blood glucose level was 70 mg/dl. The baby was exclusively breastfed starting on day 3 and was in generally good condition. However, blood glucose level was between 218 and 263 mg/dl at 5 day of age. At the age of 20 day, his blood glucose level was still high (205–260 mg/dl), and the infant was referred to the endocrine clinic for persistent hyperglycemia assessment. On physical examination, several dysmorphic features (triangular-shaped face, prominent forehead, ocular depression, lipodystrophy at the lumbar region) and inguinal hernia were present. The systolic and diastolic blood pressure measurements were 74 and 42 mmHg, respectively. The serum c-peptide and insulin levels were 2.83 ng/ml (normal: 1.0–3.5) and 120 μU/ml (normal: 2.8–13.5), respectively. Baseline chemistry including serum blood urea nitrogen was 15.3 mg/dl (normal: 7.0–20.0), creatinine 0.9 mg/dl (normal: 0.6–1.2), aspartate aminotransferase 38 U/L (normal: 14–40), and alanine aminotransferase 16 U/L (normal: 9–45), as well as complete blood count profile were within normal range. Urinalysis showed no glucose or ketones. There was no sign of ketoacidosis and the patient had no type 1 diabetes autoantibodies (antibodies against glutamic acid decarboxylase, islet cell, islet antigen-2, and insulin). The liver and pancreas ultrasonography revealed no structural abnormality. Echocardiography at the age of 1 month confirmed mild pulmonary stenosis and ASD secundum (2 mm) which did not require surgical intervention. Neonatal diabetes mellitus (NDM) was suspected on the basis of hyperglycemia occurring within the first month of life that lasted for >2 weeks and required insulin therapy. At age of 25 day, clinical exome sequencing was performed to identify the genetic cause of NDM.

To monitor the glycemic level, his blood glucose was measured at the beginning of each feeding session. The patient was treated with subcutaneous insulin, and blood glucose level gradually stabilized. The blood glucose levels ranged from 110–250 mg/dl during the next 10 days. An adequate glucose level was achieved at 6 weeks of age without insulin treatment. His body weight was 4.4 kg (<3rd percentile) and his length was 61.6 cm (<3rd percentile) at 10 months of age. The patient experienced no hyperglycemic episode and the glycated hemoglobin was 5.0% and insulin level 2.8 μU/ml. At 10 months of age, the patient had no teeth erupted in the oral cavity.

### Genetic Testing

**Figure 1:**
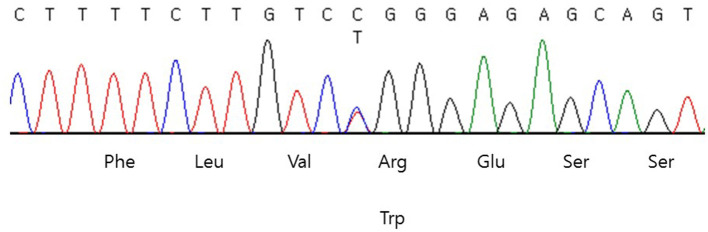
DNA sequencing chromatogram of the patient. Heterozygous missense variant, NM_181523.3:c.1945C>T (p.Arg649Trp) in exon 15 of the *PIK3R1* was identified.

Genomic DNA was extracted from peripheral blood samples using the QIAsymphony DSP DNA mini kit (Qiagen, Hilden, Germany). Sequencing was performed using a TruSight One sequencing panel (Illumina Inc., San Diego, CA, USA) consisting of 4,813 genes associated with known Mendelian genetic disorders on a NextSeq 550 (Illumina, CA, USA). Variants were assessed using the ACMG variant classification guidelines (8). Subsequently, a heterozygous missense variant, NM_181523.3: c.1945C>T (p.Arg649Trp) in exon 15 of the *PIK3R1* gene was identified (Figure 1), which was previously reported as the pathogenic variant of SHORT syndrome (4). This variant was validated using conventional Sanger sequencing. The clinical exome sequencing revealed no other pathogenic variants in genes associated with NDM (*KCNJ11, ABCC8, INS, GATA6, EIF2AK3, FOXP3*), mitochondrial diabetes (*POLG, POLG2, OPA1, RRM2B*), and maturity onset diabetes of the young (*HNF1A, GCK, HNF4A, ABCC8, BLK, HNF1B, INS, KCNJ11, KFL11, PAX4*, and *PDX1*). Segregation analysis could not be performed due to the unavailability of parental samples.

## Discussion

Herein, we report a case of TNDM in SHORT syndrome caused by a pathogenic variant in *PIK3R1*. SHORT syndrome is inherited in an autosomal dominant manner and caused by a heterozygous loss of function variant of *PIK3R1* located on chromosome 5 (5q13.1) (2). To date, fewer than 50 cases have been reported in the literature (3). Approximately 10 different pathogenic variants in *PIK3R1* have been found to cause SHORT syndrome and the c.1945C>T (p.Arg649Trp) variant was identified most frequently (2, 9). Approximately half of the patients exhibit at least four of the five characteristics that comprise its acronym. Short stature, ocular depression, and teething delay are the most common findings, with hyperextensibility of joints, and Rieger anomaly being less common (2). Our patient exhibited two of the five characteristics in the SHORT syndrome acronym (inguinal hernia, and ocular depression). His length was less than 3rd percentile and he had no teeth erupted at last visit. However, further follow-up is need to accurately the presence of short stature and teething delay because he was only 10 months old.

*PIK3R1* mutation in SHORT syndrome appears to disrupt the insulin signaling pathway and thereby predispose the patient to insulin resistance and diabetes mellitus (4–6). Of the 17 previously reported patients with c.1945C>T mutations, 9 (53%) developed diabetes mellitus and/or insulin resistance (4–6, 10, 11). The authors suggested 1945C>T (p.Arg649Trp) led to impaired interaction between p85a and IRS-1 and reduced AKT-mediated insulin signaling in fibroblasts from affected subjects (6). Insulin resistance/diabetes mellitus has also been diagnosed in patients with the other following pathogenic variants: c.1929_1933delTGGCA, c.1615_1617delATT, c.1465G>A, c.1943dupT, and c.1892G>A (5, 12).

Diabetes mellitus is common in adults with SHORT syndrome but infrequently observed in adolescents/young children, indicating that time (and/or other susceptibility factors) may be required for insulin resistance to develop (2). Our patient experienced remission of diabetes at the age of 6 week, and he is currently 10 months of age, euglycemic, and not taking any insulin or oral hypoglycemic agents. However, TNDM frequently recur in childhood, during puberty, or later in adulthood. Busiah et al. reported that remission of TNDM occurred in 51% (89/174) of patients at a median age of 17 weeks and recurrences occurred in 26% (23/89) of patients with TNDM at a median age of 12.7 years (13). When reviewing the literature to date, TNDM in SHORT syndrome has not been reported. In our patient, there were no symptoms even though the glucose level was high, insulin requirements were low, and NDM duration was short. In SHORT syndrome, DM may be present in newborns and overlooked. Therefore, we recommend that clinicians should suspect SHORT syndrome for newborns with NDM, IUGR, and facial gestalt. Genetic diagnosis of SHORT syndrome is important for an appropriate treatment and genetic counseling in affected families. Insulin resistance/diabetes mellitus, Rieger anomaly/glaucoma, dental anomalies, and hearing loss can often be treated by appropriate medical specialists if present. Clinical exome sequencing allowed us to simultaneously evaluate many genes associated with early onset diabetes mellitus. Uniparental disomy of chromosome 6 (UPD 6) is one of the important genetic causes of NDM, but it could not be evaluated by clinical exome sequencing.

IUGR is commonly observed in cases of TNDM because insulin acts as a fetal growth hormone. Consequently, insulin insufficiency coupled with the failure of transplacental insulin delivery causes newborns with TNDM to be born small for their gestational age (14). Reduced insulin signaling may also limit intrauterine growth because individuals with SHORT syndrome are usually very small at birth (<3rd percentile) and varying degrees of short stature are usually present throughout childhood (2). Despite the short stature of the patients, growth hormone therapy is not recommended for SHORT syndrome patients because of the potential to promote the development of diabetes mellitus and poor responses. Conversely, in a recent study, pubertal development and/or advanced age rather than growth hormone therapy was shown to play a critical role in the development of insulin resistant diabetes mellitus (IRDM) (11). The usefulness of an SGLT2 inhibitor and metformin in the management of IRDM in SHORT syndrome was reported (10, 11). However, in a recent study, metformin treatment was shown to paradoxically lead to a potential deterioration of insulin resistance and development of glucose intolerance in SHORT syndrome patients (15). Further studies are warranted to confirm the clinical benefits of oral hypoglycemic agents for the treatment of genetic syndromes of insulin resistance.

To the best of our knowledge, this is the first case report of SHORT syndrome with TNDM. Our findings provide important information that TNDM with insulin resistance can be a phenotype of SHORT syndrome. Familiarization and discernment of complicated genetic syndromes can enable early implementation of appropriate management and proper care of the patient.

## Data Availability Statement

The data presented in this study are available on request from the corresponding author.

## Ethics Statement

The studies involving human participants were reviewed and approved by Incheon St. Mary's Hospital Institutional Review Board. Written informed consent to participate in this study was provided by the participants' legal guardian/next of kin. Written informed consent was obtained from the minor(s)' legal guardian/next of kin for the publication of any potentially identifiable images or data included in this article. Informed consent was obtained from the patient's parents for the publication of this case report.

## Author Contributions

S-HK, D-HJ, and MyK wrote the manuscript. JY and MyK analyzed the genetic study. All the authors contributed to the manuscript.

## Conflict of Interest

The authors declare that the research was conducted in the absence of any commercial or financial relationships that could be construed as a potential conflict of interest.
